# Evaluation of a novel robotic testing method for stability and kinematics of total knee arthroplasty

**DOI:** 10.1002/ksa.12516

**Published:** 2024-10-24

**Authors:** Sander R. Holthof, Mick Rock, Richard van Arkel, Angela Brivio, David Barrett, Andrew A. Amis

**Affiliations:** ^1^ Biomechanics Group, Mechanical Engineering Department Imperial College London London UK; ^2^ De Puy Synthes Ltd Beeston Leeds UK; ^3^ King Edward VII Hospital London UK; ^4^ Department of Trauma and Orthopaedic Surgery Istituto Clinico Città Studi Milano Italy; ^5^ School of Engineering Sciences University of Southampton Southampton UK

**Keywords:** instability, kinematics, prosthesis, replacement, robotic testing, total knee arthroplasty

## Abstract

**Purpose:**

This work developed a novel preclinical test of total knee replacements (TKRs) in order to explain TKR instability linked to patient dissatisfaction. It was hypothesized that stability tests on the isolated moving prostheses would provide novel comparative data on the stability and kinematics among TKR designs.

**Methods:**

Three TKR designs, DePuy Synthes Attune MS, Stryker Triathlon and Zimmer Biomet Persona MC, were assessed using a robotic arm while flexing–extending 0–140°. Tests imposed 710 N body weight combined with three tibial loads: no anterior–posterior (AP) force, 90 N anterior or 90 N posterior force. Other load effects were minimized and the kinematics was recorded. Each implant was tested six times to investigate the repeatability of the method. Data were analysed using statistical parametric mapping with one‐way analysis of variance (ANOVA). If significance was found (*p* < 0.05), post hoc *t* tests with Bonferroni correction were used to contrast groups.

**Results:**

Significant differences were found throughout flexion–extension. Femoral rollback, AP stability, coupled internal–external rotation and AP position (roll‐back) were all influenced by implant design. AP stability of the TKRs reduced with flexion reaching Attune 15 mm, Persona 13 mm and Triathlon 21 mm at 140° flexion. Tractive rolling significantly affected kinematics in the less congruent Triathlon design, with 6 mm different paths between flexion and extension motion (*p* < 0.05 across 5–100°). Paradoxical anterior femoral sliding in early flexion (0–40°) occurred in Persona and Triathlon designs.

**Conclusions:**

The novel testing technique provides, for the first time, comparative data on the inherent stability and kinematics of the TKR implants themselves across the arc of flexion–extension, independent of variables including soft tissue behaviour and surgical technique. The data show how much each prosthesis can contribute to the stability and motion of the implanted knee. Similar data from a wider range of designs will enable more informed decisions regarding implant design choice, aiming to reduce the prevalence of TKR instability in patients.

**Level of Evidence:**

Controlled laboratory study.

**Abbreviations**
ACL: anterior cruciate ligament
ANOVA: analysis of variance
AP: anterior–posterior
ASTM: American Society for the Testing of Materials
ATT: anterior tibial translation
DOF: degrees of freedom
MC: medially congruent
MS: medially stabilized
PE: polyethylene
PMMA: polymethylmethacrylate
PTT: posterior tibial translation
SD: standard deviation
TEA: trans‐epicondylar axis
TKA: total knee arthroplasty
TKR: total knee replacement

## INTRODUCTION

Although total knee arthroplasty (TKA) is in widespread use, some patients are dissatisfied with the outcome of their knee replacement [9]. One reason for this is instability causing loss of function, pain and revision surgery [5, 20, 21, 25]. Instability post‐TKA arises from a combination of loading, patient factors, surgical technique and implant design [2, 3, 11]. Clinical studies encompass all these factors, so other test methods are required to isolate the role of the implant design—how much does the implant contribute to the stability of the implanted knee?

Studies of ‘mid‐flexion instability’ post‐TKA, both computational and fluoroscopic, have hypothesized that one reason for it is that sudden changes in the radius of the bearing surface of the femoral component in traditional multiradius total knee replacement (TKR) designs could cause a change in kinematics with a decrease in anterior–posterior (AP) stability, which could lead to the patient having a sensation of instability in mid‐flexion [4, 12, 16].

The American Society for the Testing of Materials (ASTM) standard ASTM F1223‐20 [1] sets out guidelines for AP stability testing of TKRs. The key points of this standard are:

- Testing is at 0°, 15°, 90° and maximum flexion (as defined by the manufacturer);
- For AP testing, the implant is loaded until dislocation is imminent, a mechanical stop is reached or a dangerous situation occurs;
- An axial compressive force of 710 N (the ASTM definition of body weight) is used.

However, it does not capture coupled internal/external tibial rotation which occurs with uneven medio‐lateral force distribution and with asymmetrical designs [10]. Tests at a fixed angle of knee flexion cannot capture femoral rollback, stability changes at other angles of flexion or differing flexion–extension paths resulting from tractive rolling, where the femur rolls on the tibia until it slips when the constraint overcomes the friction [24]. These limitations imply that ‘mid‐flexion instability’ (reported clinically as a feeling of the knee ‘giving way’ in activities such as descending stairs) may not be discernible with the ASTM test between 15° and 90° flexion. The development of novel implant stability tests may lead to a better understanding of implant behaviour.

This study aimed to measure the stability of the isolated TKR components, anticipating that a novel robotic test that measured their AP stability and secondary motions throughout the loaded flexion–extension cycle would be able to more completely show the effects of their design.

It was hypothesized that the novel test method would be able to show significant differences between the TKR designs regarding their femoral rollback kinematics, AP stability and coupled internal/external rotation when AP force was applied in asymmetrical designs.

## METHODS

**Figure 1:**
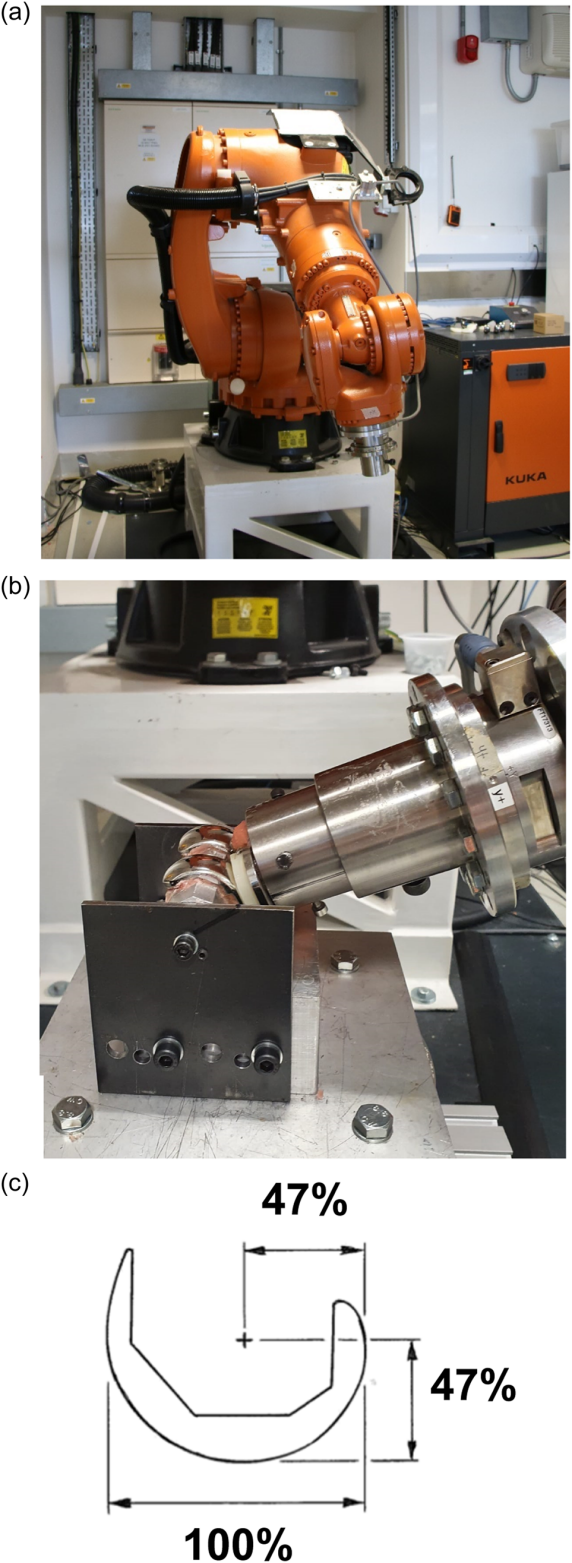
(a) Six‐degrees of freedom (DOF) robotic actuator. (b) Experimental testing set‐up with the total knee arthroplasty (TKA) at 0° flexion. The tibial component is moved around the fixed femoral component by the robot arm, to which it is attached via the 6DOF load cell at the top right of the picture. The femoral component is mounted by bolts on the transepicondylar axis. (c) In the absence of the femur, the position of the trans‐epicondylar axis was defined as being 47% of the anterior–posterior (AP) width of the medial femoral condyle from both the distal and posterior aspects.

A robotic testing method was developed (Figure 1a,b) that could measure the (1) neutral path of motion (no tibial AP translation force) of the loaded joint across the flexion/extension cycle, (2) effect of tibial AP forces on kinematics (similar to laxity tests done in anterior cruciate ligament (ACL)‐injured knees), (3) kinematics in flexion versus extension and (4) coupled internal/external rotation when an AP force is applied.

The TKRs were tested in a KUKA‐KR‐160‐R150 robotic actuator (KUKA) with ATI Delta 6 DOF force/torque load cell (ATI), controlled by SimVitro software (SimVitro), allowing force and displacement control of the implant within the knee joint‐coordinate system (Figure 1a,b). The joint coordinate system was set up per Grood and Suntay [8], with knee flexion measured around a femoral trans‐epicondylar axis (TEA). As the experiment was using only implants, the positions of the femoral epicondyles that define the TEA in a native knee were defined as in Figure 1c [23]. The TEA was parallel to the distal ‘bone cut’ surface of the mounting bar in the coronal plane, and the medial and lateral screw heads enabled the TEA to be digitized precisely (Figure 1b). The femoral mechanical axis was defined perpendicular to the TEA by digitizing around a long bolt in the centre of the femoral mounting [17]. The tibial plateau was defined using the most medial and lateral points of the rim of the tibial tray. The tibial long axis was defined perpendicular to the TEA by digitizing around the distal end of the cylindrical mounting. This kinematic setup was based on an ‘anatomical’ TEA system, as this allows comparison of the kinematics between the native knee, the isolated prosthesis (as in the present study) and postarthroplasty.

**Figure 2:**
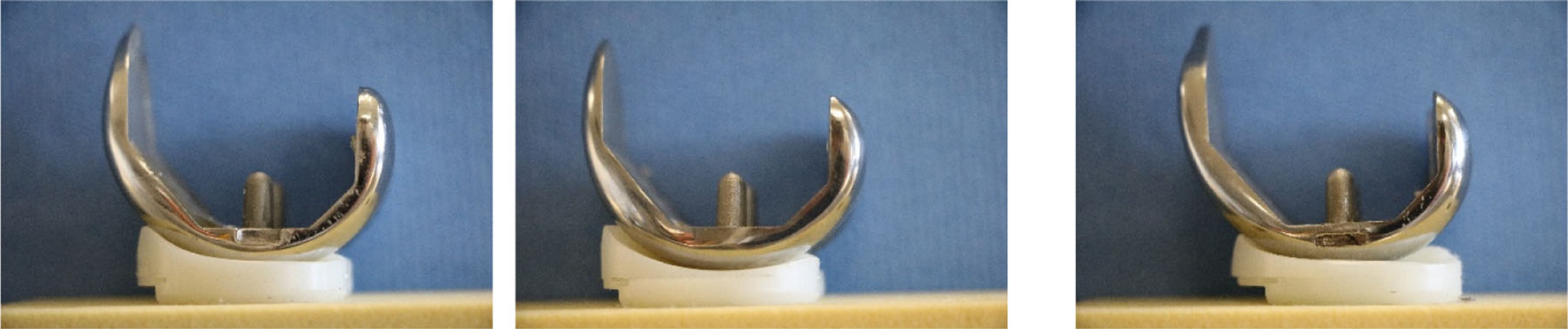
Medial view of implants tested (from left to right): Attune MS; Persona MC; Triathlon.

The implant designs tested were the Attune Medially Stabilized (MS) size 6(DePuy‐Synthes), Persona Medially Congruent (MC) size 8 (Zimmer‐Biomet) and Triathlon size 5 (Stryker) (Figure 2). All implants were the average size used clinically and had comparable AP sizes but different articular geometries.

The Attune MS had gradually reducing femoral radius from 5° to 65° flexion, then constant radius until 120° and a deep flexion radius beyond 120°. The polyethylene (PE) tibial insert was asymmetrical, being more congruent medially [6]. The Persona MC femoral geometry, sometimes known as a ‘J‐curve’, had several constant radii engaging at different angles of flexion, with smaller radii as flexion increased [19]. The medial tibial articulation was more congruent than the lateral. The Triathlon had a single radius from 10° to 110° flexion and then a smaller radius. The femur and tibia were medial‐lateral symmetrical and less congruent than the other designs tested (Figure 2c) [22].

The femoral component was cemented onto a custom fixture using polymethylmethacrylate (PMMA), attached rigidly to the robot base. The tibial component was cemented into an aluminium cylinder attached to the load cell on the moving robot end effector (Figure 1b). Pointed screws locating into the fixtures ensured precise repositioning between tests. Full extension—0° flexion—was when the distal femoral bone cut was parallel with the tibial tray. (This definition corresponds to 5–7° native knee flexion due to the posterior slope of the tibial bone cut.)

An Optotrack Certus optical tracking system (NDI) was used to digitize points representing the medial and lateral epicondyles and tibial plateau, proximal femur and distal tibia. The implant was moved into the estimated neutral (low‐point equilibrium) starting position where joint forces and torques were zero, except for 100 N axial compression, at 0° flexion. A sinusoidal 10 N AP force was applied, with three repeats, to find the ‘true’ neutral position allowing for friction in opposing directions. The compression force was then increased to 710 N (the ‘body force’ used by ASTM for TKR testing) [1]. All kinematics are reported relative to this final ‘neutral position’.

To investigate mid‐flexion instability, a novel dynamic test was used. The implant was flexed from 0° to 140° and extended back to 0° at 1.5°/s while maintaining 710 N body weight and minimizing other loading on the tibia, thus finding the ‘neutral’ path of motion. Following this, 90 N anterior tibial translation (ATT) force was also applied and the test was repeated. Lastly, 90 N posterior tibial translation (PTT) force was applied in addition to the 710 N compression and the test was repeated. The difference between the ATT and PTT curves was defined as the AP stability (actually the AP laxity) and displayed graphically with the neutral path as the zero axis. The neutral paths in flexion and extension were used to calculate the anterior and posterior laxities in flexion and extension, respectively, to account for differences resulting from tractive rolling. Each test was repeated three times, and the final flexion/extension cycle was analysed.

Each implant was tested six times to investigate the repeatability of the method. For each test, the implants were removed from the robot and fixation pots, recemented, redigitized and a new neutral point was found.

### Statistical analysis

An a priori power analysis was not performed in view of the assumption of high precision of robotic testing of isolated implants; this assumption was tested during the work.

Results were analysed using statistical parametric mapping, which allowed analysis over the continuous flexion field (http://www.spm1d.org). One‐way analysis of variance (ANOVA) was used to identify the arcs of flexion–extension with significant differences, with *p* < 0.05. If significance was found, post hoc *t* tests with Bonferroni correction were used to find differences between groups. A *t* test was used to compare stability at different flexion angles, such as before and after the flexion angle where the femoral radius changed.

Four variables were investigated:

- Neutral path motion: the AP position of the tibia relative to the femur with no AP force applied.
- Anterior–Posterior loaded position: the AP position when an ATT or PTT force was applied. The loaded position does not have the neutral path subtracted.
- Anterior–posterior laxity (the inverse of stability): the changes in AP position from the neutral path when ATT or PTT force was applied.
- Coupled tibial internal–external rotation, caused by the anterior or posterior force imposed on asymmetrical components.

## RESULTS

**Figure 3:**
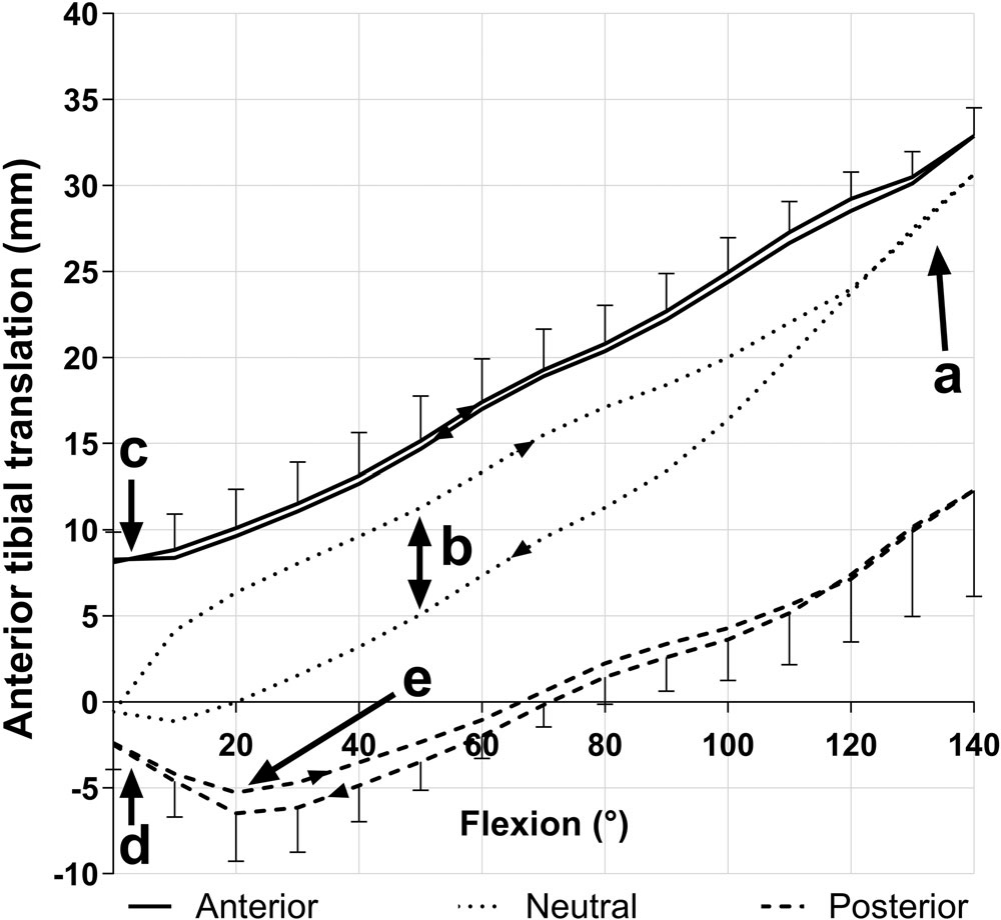
Data resulting from tests on the Triathlon design. The (0,0) origin of this graph is the initial point on the neutral path when loaded at full extension and all other points are measured relative to this. Among the features are (a) increasing anterior tibial translation of the neutral path with knee flexion, that is femoral roll‐back; (b) different paths of motion during knee flexion and extension, caused by tractive rolling; offsets arising from either anterior (c) or posterior (d) tibial translation forces that show knee prosthesis laxity/stability; (e) paradoxical posterior tibial translation (that is: femoral anterior sliding) in early flexion when a posterior tibial translation force is applied. (Mean +/– SD, *n* = 6 repeats).

An overview of the results from testing the Triathlon is shown in Figure 3:

(a) The neutral path with knee flexion shows increasing ATT, reaching 31 mm of femoral roll‐back by 140° flexion;
(b) There were different paths of motion during flexion and extension. The neutral paths were significantly different between flexion and extension across 5–100° flexion (5.9 mm mean difference), with the femur rolling relatively posteriorly in flexion (giving ATT) and anteriorly during extension—a tractive rolling effect caused by the friction in the prosthetic bearing. This effect was not found with the Attune or Persona prostheses, which had more conforming bearings.
(c) Adding 90 N anterior tibial drawer force caused 8.0 mm mean ATT laxity in extension, decreasing to 2.4 mm at 140° flexion;
(d) Adding 90 N posterior tibial drawer force caused PTT laxity that varied from 2.5 mm in extension to 18.2 mm at 140° flexion;
(e) Paradoxical PTT in early flexion: this is the femur sliding anteriorly when it might be expected to be rolling posteriorly: the femur slid anteriorly by 5.2 mm across 0–20° flexion. Similarly, in terminal knee extension, there was a paradoxical posterior femoral translation of 6.5 mm under the influence of the 90 N posterior tibial force. The Persona design also showed paradoxical femoral anterior translation up to 3.1 mm across 0–40° flexion with posterior tibial loading.

### AP stability

The difference between the graphs for ATT and PTT drawer forces gives the AP stability envelope across 0°–140° flexion.

**Figure 4:**
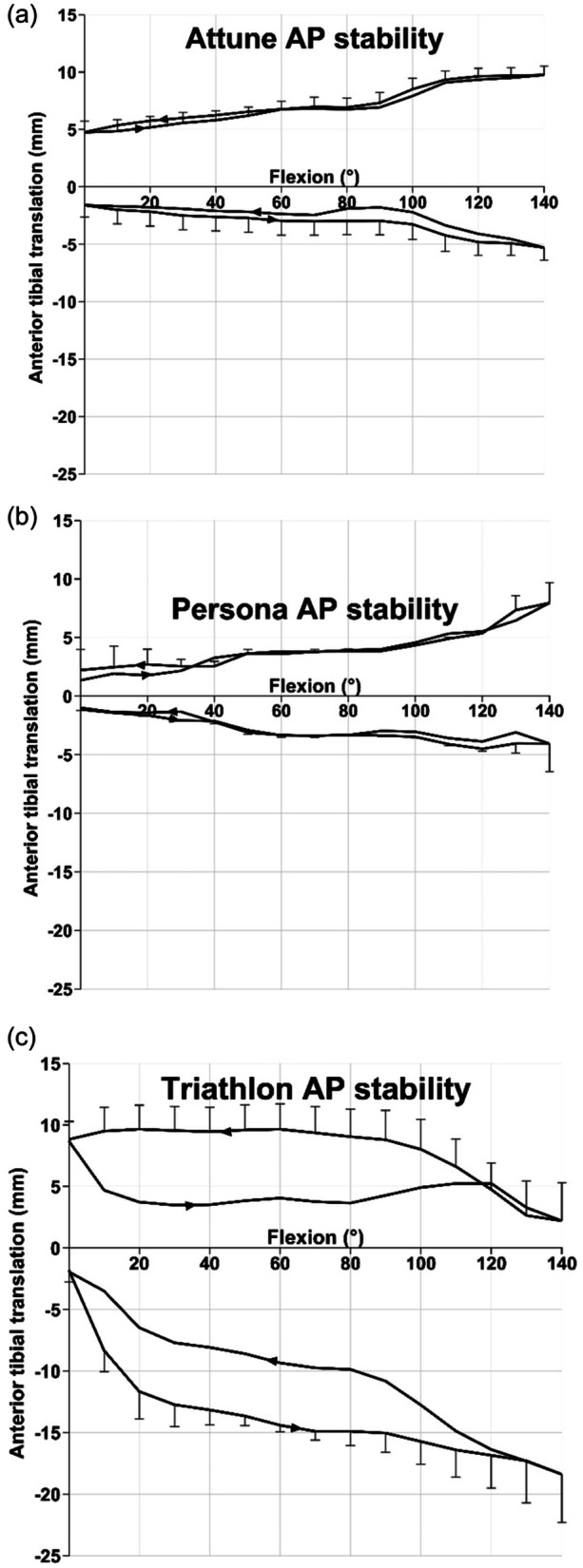
Anterior–posterior stability for (a) Attune MS; (b) Persona MC and (c) Triathlon designs. The anterior tibial translation is the difference from the neutral path of motion. In each graph, the upper sector is with the 90 N anterior tibial translation force applied and the lower is with the 90 N posterior tibial translation force applied. AP, anterior–posterior.

The Attune AP stability envelope increased from 6.3 mm at 0° flexion to 15.0 mm at 140° flexion (Figure 4a). The anterior laxity was greater than the posterior across the arc of flexion: 9.5 mm anterior and 5.5 mm posterior laxity at 140° flexion. Both ATT and PTT increased significantly, by 2.7 and 2.2 mm, around 95° flexion.

The Persona AP stability envelope was symmetrical about zero across 0–90° flexion, increasing from 2.3 mm in extension to 12.7 mm by 140° flexion when the ATT was 8.4 mm and the PTT was 4.3 mm (Figure 4b). Both anterior and posterior translations increased significantly (increased laxity/reduced stability) around 35° and 100° flexion (Figure 4b). These stability changes likely reflect transitions between arcs of differing radii of the femoral component of the Persona but not for the Attune where the radius change is in high flexion at 120°.

For the Triathlon, anterior tibial force caused 8.0 mm mean ATT in extension, decreasing to 2.4 mm in flexion. Conversely, the PTT varied from 2.5 mm in extension to 18.2 mm in flexion. Thus, the Triathlon AP stability envelope increased from 10.5 to 20.6 mm across 0–140° flexion (Figure 4c). This reflected the lower congruence of the bearing in this design (Figure 2).

### Coupled internal–external rotations

**Figure 5:**
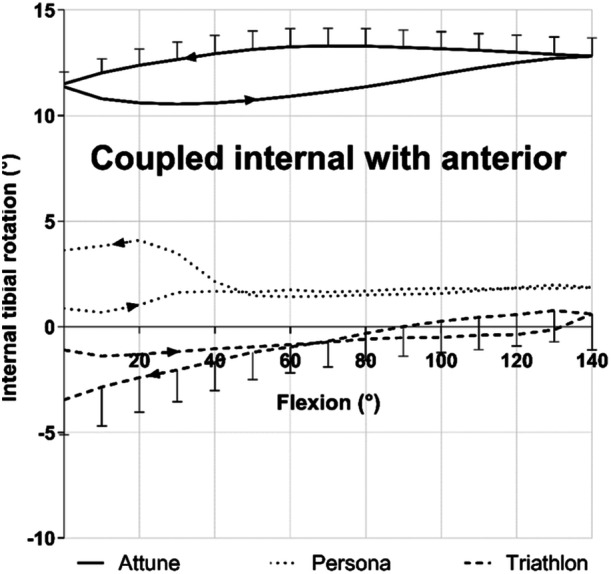
Coupled internal rotation with 90 N anterior tibial force across 0–140° knee flexion–extension.

**Figure 6:**
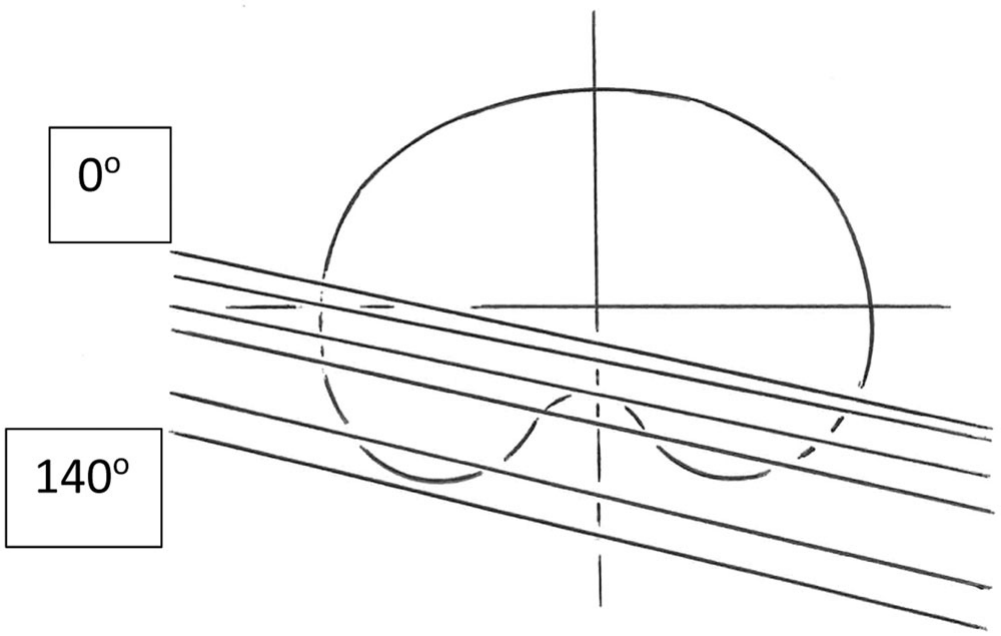
Tibial tray of Attune MS, right side, with femoral epicondylar axis superimposed at 0°, 30°, 60°, 90°, 120° and 140° knee flexion when the tibia was loaded by 710 N axial compression plus 90 N anterior translation force, which caused a coupled internal rotation of 11–13°. The anterior tibial translation increased progressively with knee flexion from 5–29 mm.

The Attune MS showed a coupled internal rotation of 11–13° with anterior tibial force (Figure 5) and ±2° external rotation with posterior tibial force. These coupled motions were expected in this asymmetrical design with a greater constraint of the ‘medially stabilized’ condyle. The data for ATT and coupled internal rotation were combined to show the relatively constant internal rotation while the femur rolled posteriorly with knee flexion (Figure 6).

The Persona MC design showed 2.1° mean coupled internal rotation with anterior tibial force across 40–140° flexion, varying between 1° and 4° across 0–40° flexion (Figure 5). With posterior tibial force the mean coupled external rotation was 1.3° before 40° flexion then a mean coupled internal rotation of 2.1° was found above 40° flexion. Larger coupled rotations had been anticipated with this 'medially congruent' design.

The Triathlon showed little coupled rotation (±2°) with anterior or PTT forces, other than near knee extension with anterior force applied, which caused a mean 3.5° external tibial rotation (Figure 5). The lack of coupled internal–external rotation arose from the symmetry of the medial and lateral condyles.

### Implant comparisons

Significant differences between implant designs were found in neutral path motion, anterior and posterior loaded positions and coupled tibial rotation during the flexion/extension cycle (Table 1).

**Table 1:** Overview of significant differences between the three implant designs for the translations and coupled rotations.

	Flexion			Extension		
Implants	Flexion angle (°)	Mean AP difference (mm)	*p* Value	Flexion angle (°)	Mean AP difference (mm)	*p* Value
***Neutral path***						
Attune—Persona	20‐47	2.2	0.006	8‐49	1.6	<0.001
	94–108	−2.8	0.007	89–110	−3	0.001
Triathlon—Attune	4–140	9.1	<0.001	51–140	−7.5	<0.001
Triathlon—Persona	4–140	8.8	<0.001	25–140	−5.6	<0.001
***Anterior path***						
Attune—Persona	0–79	4.5	<0.001	65–69	3.1	0.016
Triathlon—Attune	23–140	6.4	<0.001	7–140	−6	<0.001
Triathlon—Persona	0–140	8.9	<0.001	22–140	−8.1	<0.001
***Posterior path***						
Attune—Persona	32–55	2^a^	0.003	22–60	2	<0.001
	92–119	−2.8	0.002			

	Flexion				Extension		
**Implants**	Flexion angle (deg)	Mean internal rotation (°) difference		*p* Value	Flexion angle (deg)	Mean internal rotation (°) difference	*p* Value
***Coupled internal rotation with anterior loading***						
Attune—Persona	0–50	7.5		0.015	14–26	6.9	0.017
Attune— Triathlon	0–140	8.1		<0.001	0–140	8.8	<0.001
Persona— Triathlon	64–99	3.7		0.009	33–77	4.6	0.008
***Coupled external rotation with posterior loading***							
Attune— Persona				N/A	48–66	4.6	0.016

*Note*: The mean difference is the average of the differences across the arc of flexion where a significant difference was found. Anterior tibial translation and Internal tibial rotation are defined as positive (+), and posterior tibial translation and external tibial rotation are defined as negative (–).

^a^ During flexion with a PTT load, the path of the tibia of the Persona was 2 mm posterior to the path of the Attune (due to paradoxical motion of the Persona) across 32–55°, and it was anterior across 92–119° due to femoral rollback. These significant differences in the paths of motion are not the laxities seen in Figure 4, which reference the neutral paths.

The neutral path of the Triathlon showed higher femoral rollback (*p* < 0.001 across 4–140° flexion) than the Persona MC (8.8 mm mean difference) and Attune MS designs (9.1 mm mean difference). There were small but significant differences in neutral path motion between the Persona MC and Attune MS (Table 1). Similar significant differences among the designs persisted when the knee moved with anterior tibial force but were mostly insignificant with a posterior tibial force (Table 1).

AP tibial forces caused coupled tibial rotations that were significantly larger in the Attune design than with either the Persona or Triathlon (Table 1). This was clearer for anterior forces than for posterior, presumably because the posterior force on the tibia led to restraint of rotation caused by the femoral condyles reaching the larger anterior upward slope of the PE bearing surface in all three designs (Figure 1).

## DISCUSSION

This work shows that a novel method for testing the stability of TKR components, by applying anterior/posterior translation forces during loaded flexion–extension motion, provides a view of the behaviour of the implants that is not available from stability testing at fixed angles of flexion. As was hypothesized, the three implant designs showed significant differences in their femoral rollback kinematics, AP stability and coupled internal/external rotations. The clinical implication of these findings is that they provide the surgeon with objective data about the stability and kinematics of the TKRs themselves and thus which of them may be appropriate choices for their patients. This study has the potential to improve the understanding of prosthetic behaviour and inform the design of future implants, ultimately enhancing patient outcomes.

This study tested three widely used TKRs that behaved differently under standardized loading/motions. The data could be a starting point for a widening sample of clinically available devices, building an open‐access database. This type of information is not available to the clinician faced with choosing the most appropriate TKR design for use in routine arthroplasty and situations such as knees with partly deficient soft tissues, yet would offer objective information on which to base a choice. Even with only three ‘mainstream’ implants, the testing suggests that one of them has less inherent stability and so the clinical implication is that it would likely depend on greater soft tissue stabilization than the other two.

Static implant testing (at a fixed angle of knee flexion) is used to study TKR stability to help guide implant design. However, the current testing standard (ASTM‐F1223) [1] does not measure stability at mid‐flexion angles where patients report feelings of instability. Stability should be measured continuously across the arc of flexion to ensure that transient effects are not missed. The present study found distinct zones with AP stability changes where changes in femoral articular radius occur with the Persona design. It has been suggested that these events may be associated with patients' perceptions of instability, and the Attune design with gradually reducing radius was intended to avoid this [6, 12]. The novel test in this work, across the entire flexion cycle, provides data that appear intuitively to be more clinically relevant than fixed‐angle data.

The new test method is not intended to be a joint simulation, in which clinically derived gait dynamics are reproduced to study long‐term wear behaviour, for example. It is, instead, a practical test method that allows comparison among prosthetic designs, at an earlier stage of the development cycle. A further development of this approach would be to apply similar testing conditions to native knees in vitro and to repeat the tests post‐TKA: the resulting stability and kinematic data would indicate how well the knee behaviour postarthroplasty replicates the native knee under the same loading conditions. Comparison of the resulting data with those from the same tests on TKRs already in clinical use could help to support the introduction of novel designs to clinical trials.

A number of different approaches have been used to report kinematics and stability, including low‐point kinematics [13], TEA systems [7] and ‘functional flexion axis’ systems [26]. While these methods have been validated and are widely used, they cannot be replicated easily in isolated implant testing. Although low point data are relatively easy to get in isolated implant testing, the data are dependent on implant geometry. The method used in this article reports kinematics for implants during continuous flexion/extension in a clinically relevant way, based on the femoral TEA. The use of the TEA is constant across the experiment to allow a comparison among the implants and bony landmarks that are unaffected by TKA allow the kinematics to be related back to the behaviour of the native knee.

Both the multiradius (Persona) and single‐radius (Triathlon) implants exhibited paradoxical anterior femoral translation/sliding when a PTT force was imposed during early knee flexion. That phenomenon relates to feelings of mid‐flexion instability in patients [14, 18]. The authors are not aware of previous laboratory tests that have demonstrated this behaviour. These data may have clinical implications for knee stability post‐TKA and hence TKR choice.

The Persona was the most stable anteriorly, with the stability envelop of the Persona MC changing at flexion angles associated with traditional J‐curve radius changes. The reduced stability at the angles where the radius of the femoral component decreases is likely related to reduced articular conformity. Thus, if these radius changes happen in mid‐flexion during activities of daily living, feelings of instability might arise.

The Triathlon design exhibited the greatest femoral rollback with flexion. This is likely a consequence of the lower bearing constraint, which also led to an increase in tractive rolling, as observed by others [24]. The knee has rapid flexion–extension motion changes during the load‐acceptance phase of gait that may lead to similar anterior–posterior rolling in a low‐constraint bearing [15, 24]. The clinical implication is that the low constraint might require the soft tissues to provide more stability, with higher muscle and ligament tensions, if the sudden transient rolling effects are to be controlled.

The testing revealed significant differences among implant designs that might have clinical implications. As TKR patients become younger and more active, it is crucial to assess implants as thoroughly as possible: the test described in this work may enhance preclinical testing. This may provide valuable information for surgeons and implant designers, leading to better patient outcomes. This testing method might also be used in cadaveric work. A fundamental point is that this testing provides, for the first time, data showing the stability of the moving TKR prostheses themselves and, hence, their contribution to the overall stability of the implanted knee and how much the function of the knee must depend on the soft tissues.

### Limitations

The ‘isolated implant’ tests in this article provide contextual data on the stability of the device itself within the knee, hence providing insight into the relative contributions from the prosthesis and the soft tissues. At present, such data are lacking. No soft tissues were present in these tests: they are important for implanted knee stability, so cadaveric testing should be a further stage of preclinical implant evaluation.

Loading conditions: Constant tibial compressive and AP forces were applied. The axial compressive force was as in ASTM‐F1223 [1] and the AP forces were the same as in the clinical examination of knee stability, such as post‐ACL rupture. Loading conditions could be changed to simulate specific clinically relevant situations. Loads in vivo are also higher than the forces applied in this study [15].

Medio‐lateral force distribution: The compressive force was applied equally across the medial and lateral condyles, as in the ASTM test. During gait, the native knee is more heavily loaded in the medial compartment [15], which influences coupled rotations and stability [10]. This should be considered when testing asymmetrical implants such as ‘medial pivot’ designs.

The precision of robotic TKR stability testing: This was measured systematically during the testing by removal, dismantling, recementing and replacement into the robot for retesting. Precision was aided by placing the undersides of the components against flat faces on the mountings and using pointed screws to relocate the bone pots. The data show standard deviation (SD) of approximately 5 mm in AP translation and 7° internal–external rotation, thus showing the need for multiple tests of each implant design in order to discern significant differences—robotic tests are not immune to experimental variability.

As the testing involved only implants, the medial and lateral epicondyles, as well as the medial and lateral tibial points, were approximated by points on the test setup that enabled precise digitization and kinematics were calculated based on these consistent landmarks. While knee kinematics are susceptible to crosstalk depending on the points chosen, the functional flexion axis closely matches the TEA, which is why this joint coordinate system was chosen to report knee kinematics [7, 26].

### Conclusion

The novel testing technique provides, for the first time, comparative data on the inherent stability and kinematics of the TKR implants themselves across the arc of flexion–extension, independent of variables including soft tissue behaviour and surgical technique. The data show how much each prosthesis can contribute to the stability and motion of the implanted knee. Similar data from a wider range of designs will enable more informed decisions regarding implant design choice, aiming to reduce the prevalence of TKR instability in patients.

## AUTHOR CONTRIBUTIONS

Sander R. Holthof was involved in experimental work, data analysis and paper writing. David Barrett and Andrew A. Amis were involved in experimental work, paper writing and review of the manuscript. Mick Rock, Richard van Arkel and Andrew A. Amis contributed to the formulation of the project plan, project management, paper writing and review of the manuscript.

## CONFLICT OF INTEREST STATEMENT

S.R. Holthof is supported by a research grant from Imperial College funded by De Puy Synthes Ltd. M. Rock is an employee of De Puy Synthes Ltd. R. van Arkel and A.A. Amis are grant holders at Imperial College. D. Barrett is a consultant to De Puy Ltd.

## ETHICS STATEMENT

Research Ethics Committee approval was not required for this work.

## Data Availability

The data presented in this study are available on request via the corresponding author.
